# Use of modified diets to prevent aspiration in oropharyngeal dysphagia: is current practice justified?

**DOI:** 10.1186/s12877-018-0839-7

**Published:** 2018-07-20

**Authors:** Shaun T. O’Keeffe

**Affiliations:** 1 Department of Geriatric Medicine, Galway University Hospitals, Galway, Ireland; 2 Unit 4, Merlin Park University Hospital, Galway, Ireland

**Keywords:** Modified diet, Thickened fluid, Aspiration, Quality of life, Ethics

## Abstract

**Background:**

Although modifying diets, by thickening liquids and modifying the texture of foods, to reduce the risk of aspiration has become central to the current management of dysphagia, the effectiveness of this intervention has been questioned. This narrative review examines, and discusses possible reasons for, the apparent discrepancy between the widespread use of modified diets in current clinical practice and the limited evidence base regarding the benefits and risks of this approach.

**Discussion:**

There is no good evidence to date that thickening liquids reduces pneumonia in dysphagia and this intervention may be associated with reduced fluid intake. Texture-modified foods may contribute to undernutrition in those with dysphagia. Modified diets worsen the quality of life of those with dysphagia, and non-compliance is common. There is substantial variability in terminology and standards for modified diets, in the recommendations of individual therapists, and in the consistency of diets prepared by healthcare staff for consumption. Although use of modified diets might appear to have a rational pathophysiological basis in dysphagia, the relationship between aspiration and pneumonia is not clear-cut. Clinical experience may be a more important determinant of everyday practice than research evidence and patient preferences. There are situations in the management of dysphagia where common sense and the necessity of intervention will clearly outweigh any lack of evidence or when application of evidence-based principles can enable good decision making despite the absence of robust evidence. Nevertheless, there is a significant discrepancy between the paucity of the evidence base supporting use of modified diets and the beliefs and practices of practitioners.

**Conclusion:**

The disconnect between the limited evidence base and the widespread use of modified diets suggests the need for more careful consideration as to when modified diets might be recommended to patients. Patients (or their representatives) have a choice whether or not to accept a modified diet and must receive adequate information, about the potential risks and impact on quality of life as well as the possible benefits, to make that choice. There is an urgent need for better quality evidence regarding this intervention.

## Background

Oropharyngeal dysphagia is common in older people and in those with neurological and neurodegenerative diseases [1–6]. Dysphagia increases the risk of aspiration, or entry of food and fluid into the airways below the true vocal folds, and of aspiration pneumonia. Use of modified diets to try to prevent aspiration and its consequences in those with dysphagia has increased dramatically in recent decades [3, 7]: thickened fluids are used in up to a quarter of long-term care residents [8]; and 15–30% of long-term care residents and 30–45% of older people in acute and rehabilitation wards receive modified-texture food [9]. Dietary modifications are often guided by an instrumental assessment, such as a videofluoroscopic swallowing study (VFSS), during which the effects of physical manoeuvres and of swallowing liquids and solids of varying bolus sizes and consistencies on the passage of material through the vocal cords into the airways are examined [4].

The clinical effects of modified diets in adults with dysphagia, or in selected subgroups, have been examined in multiple high-quality systematic reviews in recent years [10–24]. These have noted the generally small size and low quality of studies and the paucity of randomized controlled trials (RCTs). A systematic search of the literature since the most recent such review [10], and using the same search strategy, did not find any relevant RCT published between May 2016 and May 2018. In the absence of new RCT evidence, it is arguable that an additional systematic review is unnecessary. Instead, this narrative review has the following goals:

- To summarise the evidence regarding the potential benefits and hazards of modified diets in dysphagia;
- To examine whether current practice regarding modified diets reflects and is supported by the current evidence base;
- To explore the reasons for any disconnect between evidence and practice; and
- To provide recommendations and suggestions regarding the appropriate use of modified diets.

## Discussion

### Modifying diets to prevent pneumonia

Overall, there is no convincing evidence to suggest that texture modified foods and thickened fluids benefit adults with dysphagia by preventing pneumonia and its consequences [10–28]. There have been few RCTs regarding the effects of thickening fluids and none regarding texture modified food. This contrasts with reasonable evidence suggesting benefits from swallow rehabilitation [29], including a large single-site RCT showing that a standard programme of early behavioural swallowing intervention (including active therapeutic approaches and dietary modification) from speech and language therapists (SLTs) in acute stroke patients reduced the risk of chest infections over six months compared with ‘usual care’ (relative risk reduction 0.56) [30]. (Usual care in this study involved patient management by the attending physicians and referral to SLTs or for a VFSS only if the physician considered it to be appropriate).

The best evidence (based on the GRADE system of rating quality of evidence [10, 31]) regarding the effects of thickened liquids in dysphagic patients on prevention of pneumonia comes from a large multi-centre study [32, 33]. Initially, Logemann et al. studied 711 patients with dementia or Parkinson’s disease who aspirated on thin liquids during a VFSS and who all received three interventions - chin-down posture while taking thin fluids and nectar-thickened and honey-thickened liquids in a head-neutral position – in a randomised order during the VFSS [32]. Half the participants aspirated on all and 25% did not aspirate on any of the interventions; 68% aspirated on chin-down, 65% on nectar-thickened and 53% on honey-thickened liquids (all pair-wise comparisons significant at *p* < 0.001 or more). Honey-thickened liquids were significantly less likely to prevent aspiration when presented last than when presented first in the sequence of trials, which may reflect the effects of fatigue.

In a follow-up study, Robbins et al. randomised 504 subjects who had done equally well (all interventions eliminated aspiration) or poorly (no intervention eliminated aspiration) to continue with one of the three interventions [33]. The cumulative incidence of pneumonia after 3 months was 9.8% in the chin-down group, 8.4% in the nectar-thick group and 15.0% in the honey-thick group (differences not significant). The median hospital stay because of pneumonia was 18 days in the honey-thick group compared with 4 days for the nectar-thick and 6 days for the chin-down groups, which may reflect an increased aspiration risk and greater difficulty in clearing the airways with more viscous fluids interventions [28, 34, 35]. More patients assigned to thickened liquids than those assigned to the chin-down posture developed dehydration (6% vs. 2%, *p* = 0.05). Urinary tract infection and fever were also more common in the thickened liquid groups. Ultimately, the trial ended early after a futility analysis concluded that enrolling additional participants would not change the results. The authors concluded that no definitive conclusions about the superiority of any of the tested interventions could be made.

Other studies provide indirect evidence regarding the effects of thickened fluids. In ‘free water protocols’, selected patients who aspirate thin fluids are allowed to drink water, often under supervision and after meticulous oral hygiene, in addition to thickened fluids. A recent meta-analysis of data from inpatient rehabilitation units concluded that there was low-quality evidence that this approach did not increase the risk of lung complications and may increase fluid intake and patient satisfaction [36]. Prospective and retrospective studies of the long-term outcomes of patients with aspiration or penetration on instrumental assessments have not found a significant association between dietary recommendations and occurrence of pulmonary complications or survival [37, 38].

### Potential hazards and disadvantages of modified diets

#### Poor hydration

Poor hydration is common in frail older people and, in particular, in nursing home residents where up to a half of residents in several studies have current or impending water-loss dehydration (when fluid output exceeds fluid input leading to raised serum osmolality) [39–41]. Some reports examining fluid intake have found a reduced intake in those receiving thickened fluids [42–45]. Vivienti and colleagues found that the greatest contribution to oral fluid intake in hospital patients with dysphagia was from food rather than beverages; however, even allowing for this, none of the patients in their study achieved their calculated fluid requirements unless enteral or parenteral fluids were given [44]. An Australian study noted that only 17% of health care facilities monitored thickened fluid consumption routinely although most staff recognised the risk of inadequate fluid intake in those given such fluids [46].

Fewer studies to date have examined the relationship between modified diets and biochemical indices of dehydration. In a retrospective study of acute stroke patients, any modification of solid diets or thickened liquids resulted in significantly elevated blood urea nitrogen/creatinine values at discharge compared with those not having that intervention [47]; these biochemical changes occurred after an average of only 3–4 days of receiving modified diets. In another study, 75% of residential care residents restricted to thickened liquids for oral hydration had biochemical indices showing dehydration [48].

#### Poor nutrition

The use of modified-texture foods, particularly pureed diets, contributes to the high prevalence of malnutrition in those with dysphagia, especially in long-term care residents [9, 49–52]. A reduction in food intake is common, and pureed diets are often poorer in calories, protein and micronutrients than regular diets. In a study of Canadian nursing homes, pureed menus tended to contain lower amounts of nutrients than regular texture menus, although there was variability between homes and in some the pureed menus provided the same or more nutrition than the regular texture menus [52].

#### Delayed medication absorption

Even a minimal increase in viscosity can delay medication dissolution and disintegration and hence bioavailability and this delay becomes greater with increasing viscosity [42, 53]. Absorption of Biopharmaceutics Classification System Class 1 (e.g. prednisolone) and Class 3 (e.g. atenolol, erythromycin and metformin) drugs may be particularly affected if given with thickened liquids [42]. Crushing and mixing tablets or capsules with thickened fluids delays dissolution of amlodipine, atenolol, carbamazepine and warfarin [54]. Although the possible clinical effects of such alterations in bioavailability aren’t known, they would be of particular concern for drugs, like carbamazepine and warfarin, that have a narrow therapeutic index.

#### Impact on quality of life

Half to two thirds of dysphagia patients are non-compliant with recommendations to take a modified diet; some are completely non-compliant, while others ‘cheat’ or ‘sneak’ proper food and drinks [55–57]. The obvious reason for non-compliance is that patients don’t like modified diets. Patients have even been noted to include refusal of thickened fluids in advance directives [32].

A recent systematic review of studies confirmed that modified diets worsen health-related quality of life significantly [58]. Patients using thickened fluids report less satisfaction with the drinks, with their level of thirst and with mouth cleanliness and use descriptions such as ‘vile’ and ‘awful’ [58, 59]. Some patients may give up preferred drinks rather than having them in their thickened form [60]. Food modification may have an even greater impact on quality of life than thickening of fluids, and consumers complain about the appearance, taste, and mouth-feel of pureed food [58, 61–63], and report that eating is reduced ‘to a matter of necessity and hunger’ [63].

#### Caregiver perceptions

Carers’ beliefs that modified diets are unpalatable also contribute to non-compliance. In a recent study of staff perceptions of pureed food, most had a negative view describing such food with terms like ‘tasteless’ and ‘mush’; positive comments such as ‘tasty’ and ‘aromatic’ mainly came from allied health professionals and dietary-nutrition staff [64].

Healthcare professionals and non-dysphagic patients who have sampled them report a strong dislike for thickened fluids, especially those of higher viscosity [65–68]. Gorham-Rowan and Coston reported the experience of 68 speech and language therapy students asked to consume only thickened liquids for 24 h: all but one had a negative response to the experience; common symptoms – perhaps related to underhydration - included persistent thirst, craving water, fatigue, headache and difficulty concentrating [68].

#### Costs associated with modified diets

In the USA, the total monthly costs for thickened liquids for a person have been estimated to be in the range of $174 to $289 [33, 69] In England, prescribing of thickeners costs the health service about £14 million per year [70]. Pre-prepared meals and fluids are often costlier but this may be offset by savings on staff preparation time and costs in long-stay units [69]. It has been argued that ‘*a modest reduction in pneumonia incidence* would (my emphasis) *produce an enormous economic benefit and increased survival, justifying management of dysphagia*’. [71] However, a reduction in pneumonia remains unproven and the costs from treating dehydration and its consequences [72] and from providing nutritional supplements in those with reduced oral intake due to modified diets would be equally deserving of consideration in any economic analysis.

### Modified diets in clinical practice

Usual clinical practice with regard to modified diets is characterised by a startling inconsistency at all levels: inconsistencies in national standards and practices; inconsistencies in how carers and professionals apply those standards that do exist; and inconsistencies in the recommendations and practices of individual therapists.

#### Lack of standardisation of modified diets

An international convention on terminology and definitions for texture-modified foods and thickened liquids has recently been agreed [73]. National approaches to describing thickened fluids include descriptive terms such as – in increasing thickness - nectar-like, honey-like and pudding-like, and rheological measures. The same variability is seen in labelling of modified foods [3, 9].

Even experienced SLTs are unable to reliably produce liquids to desired levels of thickness and have only moderate success in their ability to replicate those levels of thickness in later attempts [74]. There is considerable variability in the consistency of thickened fluids prepared by staff within and between hospitals [75]. It is likely that the same problems arise with in-house production of modified texture food [7].

#### Variability in clinical practice

The use of different modified diets among SLTs vary greatly [7, 76–78]. Among 145 experienced SLTs, although nearly half prescribed thickened liquids for one-fourth to three-fourths of their dysphagic patients, responses ranged from less than 5 % to greater than 90% [7]. A vignette (including viewing a VFSS) of a dysphagic stroke patient elicited 47 treatment techniques and more than 90 treatment combinations from 245 SLTs, including different approaches to modifying diet [78]. In one nursing home study, 91% of patients placed on modified diets were placed on overly restrictive diets [79]. It has also been reported that SLTs are not always involved in training other staff in how to prepare modified diets [7].

While conducting their RCT, Logemann et al. noted that some patients were receiving thickened fluids although they did not have any aspiration of thin liquids during the VFSS [32]. A failure to assess the need for, and to review the acceptability and effectiveness of, modified diets in residential care settings may result from staff shortages or inadequate funding of services provided by SLTs [80]. In an Australian survey of attitudes of different disciplines to mealtime issues in residential care, some participants reported that nurses had the skills to assess swallowing problems and to modify the texture of diets accordingly; a common response was to place residents on a puree diet and thickened fluids without specification of the degree of thickening [80]. Studies of nursing home residents and their families also suggest ‘a blanket provision’ of modified diets in some nursing homes [62].

### Why is there a disconnect between evidence and practice?

The lack of evidence supporting the use of modified diets to reduce pneumonia is widely acknowledged in the dysphagia literature, and several writers have expressed unease at how widely they are used in everyday practice despite the lack of evidence [26, 81–83]. However, even among dysphagia experts, acknowledgement that the evidence base is not strong may be followed may be followed declarations that modified diets are important to maintain health [84]. Commentators have suggested a number of reasons for the discrepancy between evidence and practice and for continuing to recommend modified diets in some circumstances (Table 1) [71, 85–89].

**Table 1:** Non-evidence-based arguments for and against use of modified diets

Argument	Pro	Con
Modifying diets has a rational pathophysiological basis.	Dietary modifications make swallowing easier and safer in those with dysphagia The risk of aspiration in instrumental studies is often reduced by dietary modification. Similar dietary modifications in everyday life might reduce aspiration pneumonia.	Many physiologically rational healthcare interventions have ultimately been shown not to benefit patients. Aspiration pneumonia is not a direct and inevitable consequence of aspiration. Modifying diets will not influence the aspiration of saliva and secretions. Instrumental swallowing assessment bears little resemblance to eating in real life.
Evidence-based decision making is not just about clinical trial evidence.	There is sometimes an overemphasis on (the lack of) trial evidence. Absence of evidence is not evidence of absence. Decision making requires the integration of the best evidence available with clinical expertise and patient values.	The absence of robust evidence is problematic because modifying diets is intrusive and carries significant possible hazards as well as benefits. Many practitioners overestimate the benefits of modified diets. There is evidence that clinical experience outweighs research evidence and patient preferences in determining everyday practice regarding modified diets.
Modified diets are justified if consent is obtained.	Patients should make their own decision after receiving adequate information about the potential benefits and risks of modified diets.	Strong beliefs that modified diets are beneficial might influence how practitioners present risks and benefits to patients.
There are exceptions to the need for research evidence	Modified diets may be required if there is an immediate and significant distress related to feeding or a high risk of asphyxiation. Modified diets may be justified if used in conjunction with an active swallow rehabilitation program. An individual treatment trial is warranted if there is reasonable expectation of benefit, the patient agrees, and there is follow-up to assess the impact of treatment.	Follow-up assessment is not always available, perhaps especially in residential care settings.

#### Modified diets have a rational pathophysiological basis in dysphagia

Modifying diets is a rational approach to reducing aspiration, and this, irrespective of any paucity of long-term evidence, may justify their use [19, 71]. Studies of the effects of bolus modification on swallow safety show that thickening liquids slows their flow rate, allowing more time for airway closure and reduces the risk of aspiration; modified texture food are easier and safer to swallow for those with chewing problems [1–4]. The avoidance of aspiration by modifying bolus consistency in a swallowing study suggests that modifying everyday dietary consistencies for that person to a similar degree might reduce their subsequent risk of aspiration pneumonia.

This argument sounds plausible, but it is only a hypothesis; many widely-used interventions in medicine that should have benefited patients, based on pathophysiological reasoning, ultimately were shown to have no benefit, or even to cause harm [90, 91]. There are also reasons to question whether modifying diets to prevent aspiration pneumonia might represent a considerable over-simplification of the issue.

- Aspiration pneumonia is not a direct and inevitable consequence of aspiration: not all who aspirate develop pneumonia, and many who develop pneumonia are not aspirators [92–95]. Dysphagia is one but not necessarily the strongest risk factor for aspiration pneumonia [92]. Colonization of the oropharynx with pathogens and subsequent aspiration of infected saliva and secretions is important in the pathogenesis of aspiration pneumonia [96]. Modifying diets will not influence the aspiration of saliva and secretions.
- An instrumental swallowing assessment bears little resemblance to eating in real life and fluids used during videofluoroscopy do not provide an accurate indication of swallowing ability at mealtime [97]. There remain concerns regarding the interrater and intrarater reliability of instrumental assessments of swallow even with highly-trained raters [98], and test-retest reliability of assessments is unknown and might be affected by factors such as fatigue [35].
- Although the risk of aspiration in instrumental studies is reduced with liquids with increasing degrees of thickness, higher viscosity liquids also result in increased pharyngeal residue after swallowing with the potential for increased aspiration risk [28]. The same may be true for some modified texture foods [25].

#### Evidence-based decision making is not just about RCTs

Other writers have criticised the over-emphasis on (the lack of) RCT evidence regarding dysphagia interventions [71, 86]. Many healthcare interventions lack good RCT evidence; this doesn’t mean they don’t work nor that they should not be used [71, 99, 100]. Evidence based practice has been defined as “the integration of best research evidence with clinical expertise and patient values” [101]. Good clinical practice is more complex than trials alone. Even when good evidence exists, it is not always generalizable to the individual patient, and clinical expertise will be needed to adapt research findings to the needs and wishes of the individual patient.

These are strong arguments. Practitioners faced with a patient with troublesome dysphagia must often make pragmatic recommendations even if based on imperfect evidence. It is also true, however, that the absence of robust evidence has a more detrimental effect on decision making if an intervention, as with modified diets, is intrusive and carries significant potential hazards as well as benefits [102, 103].

#### Attitudes and knowledge of practitioners

Surveys of practicing SLTs suggest a strong consensus supporting modified diets based primarily on therapists’ training and experience [7, 8, 87]. Quantitative and qualitative surveys of the attitudes and beliefs of practitioners suggest that clinical experience and pragmatic considerations outweigh research evidence and patient preferences in determining everyday practice [87–89]. Ignorance of the lack of evidence may be a factor: one paper commented on ‘a lack of research-based reasoning’, [87] and a survey of 145 SLTs found that 85% agreed and only 5% disagreed that thickening liquids was effective in dysphagia [7]. Also, even therapists familiar with the lack of evidence may be uncomfortable recommending thin fluids for someone who is known to aspirate [81].

#### Modified diets are justified if consent is obtained

As with other healthcare interventions, the person or, if he or she lacks capacity to decide, their representative should decide - and accept responsibility for the consequences of that decision - whether to take a modified diet after receiving adequate information about the risks and benefits of their use [32, 104, 105]. This is a persuasive argument provided that the ethical requirements of informed consent - provision of accurate and balanced information regarding treatment options and their benefits and risks and the voluntariness of choice - are met.

Given the lack of robust evidence, most recommendations to modify diets should come hedged with ‘maybe’s’ and with acknowledgements of uncertainty rather than with ‘must’s’. However, the strong belief of some practitioners that modified diets are essential and beneficial might influence how they educate patients (as well as catering, nursing and other staff) about modified diets [78, 87]. Ullrich and Crichton, in a qualitative study of the attitudes of staff and nursing home residents and their families regarding texture modified food, commented on a risk-averse culture of care: ‘Risk management was …. communicated through language that was punitive—stripped down to imperatives that attended solely to compliance…’ [63].

Some reports regarding those who are non-compliant with recommendations to modify diets raise questions about how voluntarily people give consent. Non-compliance may cause ‘moral unease or distress’ to staff; some may refuse to participate in feeding a non-compliant patient; patients who remains determined to make what is seen as an unduly risky choice may be threatened with premature discharge [106]. Non-compliant patients may be asked to sign waivers of liability [106–108], although this approach has been criticized by others [109, 110].

### Exceptions to the need for research evidence

Situations do arise in the management of dysphagia where common sense and the necessity of intervention will clearly outweigh any lack of evidence or when application of evidence-based principles can enable good decision making despite the absence of robust RCT evidence.

#### Immediate and significant distress or danger

It seems obvious that assessment and intervention is needed if there is immediate and significant distress related to feeding or a high risk of asphyxiation. (‘Immediate’ and ‘significant’ are highlighted: the distress that might result from developing pneumonia or occurrence of non-severe symptoms such as a wet voice or a clearing cough should not be used as a justification for widespread intervention). People with severe aspiration or chewing problems may be fearful of taking thin liquids and a normal diet [85]. They may, even without liking the diet, appreciate the greater control and lower risk of immediate aspiration from a modified diet.

#### In conjunction with a swallow rehabilitation program

There is some evidence of benefit from swallow rehabilitation programs, and intensive swallow therapy reduces chest infections in dysphagic acute stroke patients [29, 30]. Patients undergoing swallow rehabilitation may also, if judged appropriate, receive modified diets. This implies, even without direct evidence, that similar benefits can be expected in other populations undergoing active swallow rehabilitation. Also, modified diets seem likely to be more acceptable to patients if they are for a limited period under practitioner supervision in this context.

#### Individual treatment trial

Even in the absence of high quality evidence, a ‘carefully controlled single patient treatment trial’ to evaluate the effect of an intervention is justified if an intervention is logical for that patient, there is a reasonable expectation of benefit and the patient agrees [111]. This seems a reasonable approach when, for example, an instrumental assessment suggests that someone might benefit from a particular modified diet. It acknowledges, in particular, the importance of the patient’s perspective, the need for any discussion regarding an intervention to be dependent on the quality of evidence and the importance of follow-up.

## Conclusions

There is little good evidence of benefit from modified diets and there is the potential for harm including as adverse impact on quality of life. This does not mean that use of modified diets should be abandoned. It does suggest the need for careful consideration as to when modified diets might be recommended to patients and how discussions about modified diets should be conducted and informed consent sought.

### When should modified diets be considered or recommended?

It is likely that practitioners will have to continue to consider the appropriateness of modified diets for patients with dysphagia without the benefit of RCT evidence for the foreseeable future. (Examination of the International Clinical Trials Registry Platform (23rd May 2018) shows no registered trial dealing with this topic). It has been argued persuasively that, even in these circumstances, application of the principles of evidence-based practice can lead to practitioners and their patients making reasonable decisions about this intervention [32, 111]. Individual trials of treatment, after discussing the possible benefits and risks and their own preferences with patients, with appropriate follow-up will often be a reasonable option.

While there are no doubt many settings where such good practices are followed, some studies raise concerns about whether everyday practice and attitudes towards modified diets does reflect an evidence-based approach. These include studies showing an overreliance of some practitioners on clinical experience and training to guide practice and the provision of modified diets, perhaps particularly in long-stay units, without appropriate assessment, communication, implementation and review. It is true that some such practices are outside the control of clinicians [80]; nevertheless, clinicians should not be complicit in poor practice and should communicate and document their concerns to their patients and to the relevant institutions.

### Communicating with patients about modified diets

#### Tone of discussions and strength of recommendations

The tone of the discussions between clinicians and patients and the strength of recommendations of the former should be highly dependent on the quality of the evidence. There should be, in effect, a sliding scale of how strongly clinicians should recommend modified diets from, for example, urging or pleading with a reluctant or non-compliant patient to accept some modification of food texture if their risk of an asphyxial death is high to, at most, a tentative suggestion of a trial of therapy if there might be sufficient benefit from a thick fluid consistency to offset the risks of dehydration and impact on quality of life. Ultimately, the patient must understand that, irrespective of the view of the clinician, he or she has a choice.

#### What level of disclosure is required regarding benefits and risks

Clinician recommendations and patient decisions are based on their relative assessments of whether the potential benefits of modified diets (or other interventions) will outweigh the potential risks (including impaired quality of life). It is for the patient to decide how he or she would judge that balance. In order to make that decision the patient needs and is entitled to information, not only about the potential for reducing aspiration pneumonia, but about possible risks and hazards, including underhydration and nutrition and the impact on quality of life [112]. Uncertainty, while a potential cause of stress for patients, is a fair description of current knowledge of the potential benefits and risks from modified diets and should be acknowledged and communicated when discussing this intervention. It would be unacceptable to seek to withhold information about the possible hazards of modified fluids to try and improve adherence to clinician recommendations.

#### Quality of life

Quality of life is as or more important than quantity of life for many people [103, 113–116]. The fact that modified diets often worsen quality of life suggests that non-compliance is an understandable and rational choice for many people [58, 65]. This would still be the case even if there were evidence that non-compliance result in adverse effects. One benefit of reviewing patients who have had a trial of therapy with modified diets is that they can balance any possible benefits (such as absence of chest infections), possible risks (such as need for treatment with parenteral fluids) and their lived experience with modified diets and make a fresh, and more-informed, decision whether to continue with a modified diet.

### Need for further research

Modified diets are a more intrusive intervention than any medication and are widely used in the absence of a high-quality evidence base. Individual patient trials may be the best that can be achieved at present but are no substitute for having RCT evidence to guide practice. Such trials are even more important if widespread screening of those who do not present with worrying symptoms directly suggestive of aspiration problems is to be recommended [117]. There is also much to learn about how best to monitor those receiving modified diets, in particular for hydration status. Biochemical testing, perhaps weekly initially and then less frequently, would seem the most obvious candidate but this requires further evaluation. ‘More research is needed’ is an often-derided cliché, but it is, I believe, a justified conclusion of many reviews dealing with modified diets in dysphagia [12–20].

## Abbreviations

RCT: randomized controlled trial
SLT: speech and language therapist
VFSS: videofluoroscopic swallowing study
